# Insect transformation with *piggyBac*: getting the number of injections just right

**DOI:** 10.1111/imb.12220

**Published:** 2016-03-30

**Authors:** M. Gregory, L. Alphey, N. I. Morrison, S. M. Shimeld

**Affiliations:** ^1^ Department of ZoologyUniversity of OxfordOxfordUK; ^2^ Oxitec LtdAbingdonUK; ^3^ The Pirbright InstitutePirbrightSurreyUK

**Keywords:** piggyBac, insect transformation, moths, transposases, transposable element, genetic, DNA transposable elements genetic vectors, germ‐line mutation, Monte‐Carlo, Markov‐Chain, decision model, goldilocks, insects, microinjection, transformation efficiency, survival, embryo, sterile insect technique, fluorescent proteins

## Abstract

The insertion of exogenous genetic cargo into insects using transposable elements is a powerful research tool with potential applications in meeting food security and public health challenges facing humanity. *piggyBac* is the transposable element most commonly utilized for insect germline transformation. The described efficiency of this process is variable in the published literature, and a comprehensive review of transformation efficiency in insects is lacking. This study compared and contrasted all available published data with a comprehensive data set provided by a biotechnology group specializing in insect transformation. Based on analysis of these data, with particular focus on the more complete observational data from the biotechnology group, we designed a decision tool to aid researchers' decision‐making when using *piggyBac* to transform insects by microinjection. A combination of statistical techniques was used to define appropriate summary statistics of *piggyBac* transformation efficiency by species and insect order. Publication bias was assessed by comparing the data sets. The bias was assessed using strategies co‐opted from the medical literature. The work culminated in building the Goldilocks decision tool, a Markov‐Chain Monte‐Carlo simulation operated via a graphical interface and providing guidance on best practice for those seeking to transform insects using *piggyBac*.

## Introduction

### Insect transformation using *piggyBac*

The ability to integrate genetic constructs into the genome of organisms has utility in mitigating some of the global challenges facing humanity (Morales *et al*., 2007; Bazuin *et al*., 2011; Kim & Pyykko, 2011). Insect germline transformation (synonymous with insect transgenesis) can be employed to alter the phenotype of an insect by gene insertion (Fraser, 2012) and represents a research area attracting global interest (Tamura *et* al., 2000; Handler & Harrell, 2001; Perera *et al*., 2002; Sarkar *et al*., 2003; Morrison *et* al., 2010; Raphael *et al*., 2011).

Various methods can be employed to achieve genetic transformation. Transposable element (transposon) vector systems (Piégu *et al*., 2015) were developed for *Drosophila melanogaster* using the *P* element (Rubin & Spradling, 1982). Although *P* works in only a very limited range of insect species, similar systems using other Class II transposable elements were developed for non‐*Drosophila* insects. The most commonly used of these is the *piggyBac* element, originally discovered in cell lines of the cabbage looper moth, *Trichoplusia ni* (Fraser *et al*., 1983; Sarkar *et al*., 2003; Zimowska & Handler, 2006). It has been used for germline transformation in multiple insect orders (Table 1). A recent review by Kim & Pyykko (2011) summarized the molecular structure and mobility of *piggyBac*. The 2472‐bp‐long element is structured with two sets of inverted repeats at both ends and a central transposase‐encoding open reading frame (Fraser, 2012). The insertion site of *piggyBac* is quasi‐random, with a cut‐and‐paste insertion at the short genome motif site of TTAA (O'Brochta, 2003; Wu & Burgess, 2004; Zhuang *et al*., 2010).

**Table 1:** Summary of the earliest successful transformation of insect species using *piggyBac*. Modified from Morrison *et al*. (2010)

Family	Species name(s)	Reference
**Mosquitoes**		
Culicidae	Yellow fever mosquito, *Aedes aegypti*	(Kokoza *et al.*, 2001)
	Asian tiger mosquito, *Aedes albopictus*	(Labbé *et al.*, 2010)
	*Aedes fluviatilis*	(Rodrigues *et al.*, 2006)
	New World malaria mosquito, *Anopheles albimanus*	(Perera *et al.*, 2002)
	African malaria mosquito, *Anopheles gambiae*	(Grossman *et al.*, 2001)
	Indo‐Pakistan malaria mosquito, *Anopheles stephensi*	(Ito *et al.*, 2002; Nolan *et al.*, 2002)
**Fruit flies**		
Drosophilidae	Common fruit fly, *Drosophila melanogaster*	(Handler & Harrell, 1999)
	Spotted‐wing drosophila, *Drosophila suzukii*	(Schetelig *et al.*, 2013)
Tephritidae	Mexican fruit fly, *Anastrepha ludens*	(Condon *et al.*, 2007)
	Caribbean fruit fly, *Anastrepha suspensa*	(Handler & Harrell, 2001b)
	Oriental fruit fly, *Bactrocera dorsalis*	(Handler *et al.*, 1998)
	Queensland fruit fly, *Bactrocera tryoni*	(Raphael *et al.*, 2011)
	Mediterranean fruit fly, *Ceratitis capitata*	(Handler *et al.*, 1998)
**Other Diptera (pest, myiasis, biting flies)**		
Muscidae	Housefly, *Musca domestica*	(Hediger *et al.*, 2001)
Calliphoridae	Australian sheep blowfly, *Lucilia cuprina*	(Heinrich *et al.*, 2002)
	New World screwworm, *Cochliomyia hominivorax*	(Allen *et al.*, 2004)
Diopsidae	Stalk‐eyed flies, *Teleopsis dalmanni*	(Warren *et al.*, 2010)
**Wasps, bees and ants**		
Hymenoptera	Sawfly, *Athalia rosae*	(Sumitani *et al.*, 2003)
	Honeybee, *Apis mellifera*	Schulte *et al.*, 2014
**Beetles**		
Coccinellidae	Harlequin ladybird, *Harmonia axyridis*	(Kuwayama *et al.*, 2006)
Tenebrionidae	Red flour beetle, *Tribolium castaneum*	(Berghammer *et al.*, 1999)
**Butterflies and moths**		
Nymphalidae	Squinting bush brown butterfly, *Bicyclus anynana*	(Marcus *et al.*, 2004)
Gelechiidae	Pink bollworm, *Pectinophora gossypiella*	(Peloquin *et al.*, 2000)
Bombycidae	Silkworm, *Bombyx mori*	(Tamura *et al.*, 2000)
Plutellidae	Diamondback moth, *Plutella xylostella*	(Martins *et al.*, 2012)
Crambidae	Asian corn borer, *Ostrinia furnacalis*	(Liu *et al.*, 2012)
Tortricidae	Codling moth, *Cydia pomonella*	(Ferguson *et al.*, 2011)

For insect transformation, *piggyBac* constructs and the respective source of helper transposase are typically microinjected into preblastoderm embryos, with the offspring of the injection survivors examined for the expression of a marker gene, typically a fluorescent protein. A recent review described the transposon vectors as having an ‘experimentally effective frequency, [however] the process remains relatively laborious and time‐consuming. Frequencies on the order of 0.1% to 10% are achievable, with higher frequencies less probable than lower ones' (Fraser, 2012).

Alternatives do exist, including electroporation, ultrasonic activation and use of a ‘gene gun’ (Wells, 2004; Mehier‐Humbert & Guy, 2005; Al‐Dosari & Gao, 2009). Other transposable elements are used for insect germline transformation, and other molecular methods are available. This study was restricted to *piggyBac* as the most widely used method and correspondingly the one for which most data are available. This may provide a benchmark against which the efficiency of other methods may be compared.

Meta‐analyses of data from multiple primary studies can be used to improve the efficiency of the scientific process (Brandt *et al*., 2013) while simultaneously dispelling misconceptions (McClain *et al*., 2015). Meta‐analyses are usually associated with clinical trials and the medical literature, but recent co‐opting of this technique has proven its applicability and usefulness to other scientific disciplines (Castellanos & Verdú, 2012). Meta‐analyses facilitate the elucidation of effect sizes and interstudy variation despite noisy backgrounds associated with a typical single observational study.

Here we provide a description of the transformation efficiency of insect transgenesis using *piggyBac* as the vector. We draw upon a systematic literature analysis and an analysis of an unpublished data set provided by the biotechnology company, Oxitec Ltd. The application of the decision tools and information therein provides researchers with an approximation of what to expect when conducting insect transgenesis using *piggyBac*, complementing other attempts in the literature to characterize and quantify costs of genetic control (Alphey *et al*., 2011).

## Results and discussion

### Systematic review of transformation efficiencies in published literature

Design and implementation of the meta‐analysis followed guidelines in Khoshdel *et al*. (2006). Meta‐analyses tend to be conducted in the medical literature, so methods were co‐opted as appropriate (Reade *et al*., 2008; Cooper and Patall, 2009). The structure of the Experimental procedures section follows Sim *et al*. (2011). A checklist for evaluation of meta‐analysis quality is described by Huf *et al*. (2011). Full details can be found in the Supporting Information.

#### Data sources

**Figure 1:**
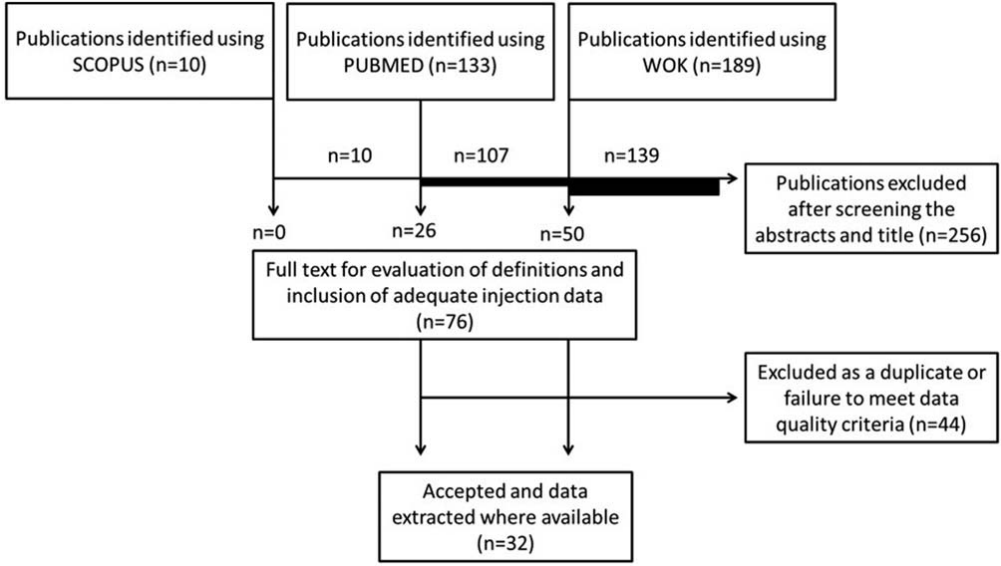
Flow chart of the paper selection process using the search term, across three life science relevant databases [SCOPUS, PUBMED and Web of Knowledge (WOK)]. 32 publications describing 86 unique experiments provided microinjection data and transformation efficiency estimates across a range of insect species.

A summary of the literature search can be seen in Fig. 1 (described further in the ‘Meta‐analysis’ section below). The following checklist was applied to candidate studies to be included in the analysis following discovery:

1. The species transformed is an insect.
2. The insect germline was transformed using the *piggyBac* vector.
3. The *piggyBac* vector was microinjected into embryos.

Injection data are included: specifically number of injected embryos, number of injection survivors and number of independent transgenic lines generated per unique construct (the derived variables; survival and transformation efficiency, were calculated from the raw data).

Typical reasons for non‐inclusion were: different methods of transgenesis; transgenesis in cell lines rather than the whole organism (for example in Mandrioli & Wimmer, 2003) and interdatabase duplication. Following this process, 32 studies remained (Fig. 1; Table 1). Additional details concerning the data extraction methods, summary statistic of choice and bias considerations are given in the Supporting Information.

### Transformation efficiency by insect order and species from published data

**Figure 2:**
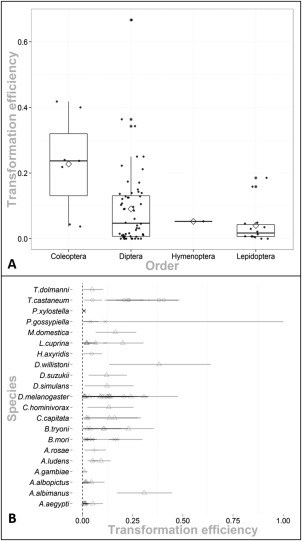
(A) A box‐and‐whisker plot of the meta‐analysis transformation efficiency data subsetted by insect order. Each subset was comprised of n equal to: Coleoptera, seven; Diptera, 52; Hymenoptera, one; and Lepidoptera, 14. The dark horizontal line represents the median, the box the interquartile range (IQR) and the whiskers 1.5 times the IQR, and the black asterisks are supplementary to the scatterplot and identify horizontally adjacent outliers within an order. The Hymenoptera and Lepidoptera do not have whiskers plotted as all non‐anomalous data are found within the IQR. A horizontal jitter plot is superimposed onto the boxplot showing the transformation efficiency of each unique construct species combination found within the literature search. The grand mean by insect order is represented by the empty diamond. (B) Published transformation efficiencies in insects found by this systematic analysis, sorted by species (alphabetical order by species). The order to which the species belongs is represented by the shape of the points (Diptera; triangle, Coleoptera; circle, Lepidoptera; cross and Hymenoptera; plus). Mean estimate from individual experiments shown by the shapes. Horizontal lines represent the upper and lower 95% confidence intervals of each experimental mean calculated using Bayesian methods (with prior distribution provided by beta distribution fitted to the combined data; shape 1 = 0.73 and shape 2 = 5.67) in R with the package ‘binom’ (Dorai‐Raj, 2014). Each experimental construct species combination has its own mean and confidence interval; the transparency of the points and intervals allows overlap to be visualized.

Germline transformation or transgenesis has been achieved across a diverse range of insect orders (Table 1). Some authors have hinted at a difference between transformation efficiencies amongst orders, with the Lepidoptera efficiencies being lower compared to the Diptera for example (Marec *et al*., 2005). The data were plotted to examine this at order level (Fig. 2A) and at species level (Fig. 2B). Most of the transformation efficiency estimates (52/74) were from transformed dipteran species, with 22 of those of the genus *Drosophila*. All medians were between 0.001 and 0.1 except for the coleopteran estimate of 0.237 (see for discussion of appropriate statistics and methods to describe the distributions). The lower whisker in the Coleoptera is the data point provided by the only non‐*Tribolium castaneum* transformed beetle; the ladybird *Harmonia axyridis* at 0.0370 (see Supplementary 5.1.3 for Bayesian methods to produce a posterior probability distribution for the transformation efficiency of a species). The Hymenoptera have only one representative so were excluded from comparison.

Outliers are a common characteristic in each order, with some transformation efficiencies of over 0.3 occurring. Following inspection it was found that the outliers were produced by less precise transformation efficiency mean estimates owing to a relatively small number of trials for certain *piggyBac*–insect combinations. For example, the lepidopteran *Bombyx mori* produced an outlier experiment with five transformed lines from 27 G_0_. As pointed out by Fraser (2012), the more extreme the transformation efficiencies the less frequently those efficiencies are observed (see Supplementary 5.1.1.3 for more detail).

### Analysis of an extensive unpublished data set of *piggyBac* transgenesis experiments

**Figure 3:**
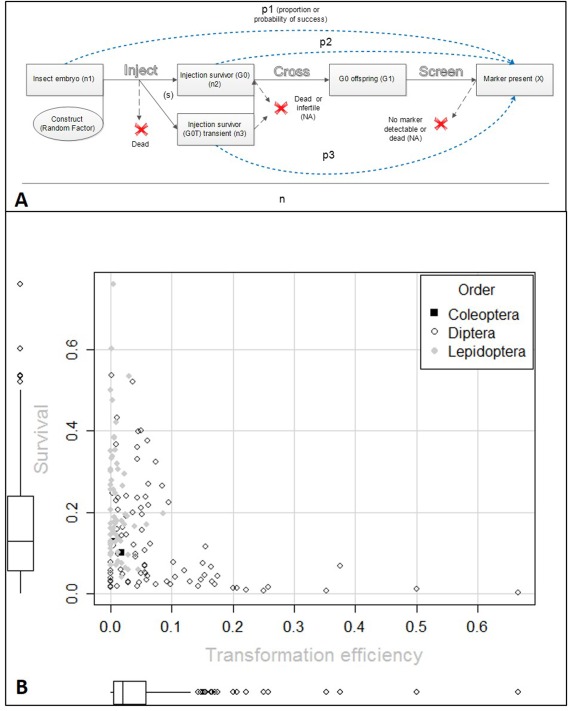
(A) The experimental unit set**‐**up of variables and statistics of interest after a given construct was injected into an embryo n_1_ times, with probability of survival s. Values not observed or recorded are annotated with ‘NA’ (not available). The outcome of each trial, n, is assumed to be independent from the outcome of all other trials. After injection the embryo has p_1_ probability of producing transgenic offspring. The embryo will either die, or develop into a transient (G_0_T) (injection survivors showing transient expression of the fluorescent marker) or nontransient fertile adult (G_0_) (injection survivor not showing transient expression of the fluorescent marker or a transient adult that was not detected as transient). Considering the G_0_ survivors, n_2_ – a proportion, p_2_, will produce at least one transgenic offspring (known as ‘transformation efficiency’ in Warren *et al*., 2010 and Martins *et al*., 2012) This particular transformation efficiency (X/G_0_ and X/n_2_) leads to a lower calculated rate than if only fertile G_0_ individuals were considered; as we have no estimate of the infertility or fertility rate in G_0_; this could not be corrected for. Multiple transgenic G_1_ from the same G_0_ parent pool are assumed to represent a single transformation event unless shown otherwise with molecular tests. Of the G_0_T, n_3_ ‐ a proportion, p_3_ will produce transgenic offspring. The proportions p_1_–p_3_ are bounded between zero and one and are derived from how many times an event, transgenesis (X), did or did not occur (the numerator). The phrases transgenic efficiency and transgenic rate are used interchangeably for p_2_ (p_1_ is not given a name despite recommendations from Warren *et al.*, 2010). (B) The correlation between microinjection survival and the achieved transformation efficiency is described. Each point represents an evaluation unit, the number of successes of a unique construct injected into a species of preblastoderm embryo, divided by the number of trials. The survival axis corresponds to number of injection survivors (to adulthood) divided by the number of embryos injected. The transformation efficiency is derived from the number of injection survivors divided by the number of independent transgenic lines generated. The figure does not group the data into species hence the more uniform and poly‐modal shape along the survival axis. A box‐and‐whisker plot on each axis describes the density of the data.

The biotechnology company Oxitec has collected a data set of over 250 000 insect injection experiments (Fig. 3A) using *piggyBac* (Table 2), more than doubling the data set used for the meta‐analysis (119 557 injections). The data were collated and subjected to exploratory data analysis of the derived variables microinjection survival and transformation efficiency to establish typical values and any discrepancy from the published data set.

**Table 2:** Summary of the data set; subsets organized by species. Information includes number of unique constructs injected into a species (the evaluation unit), sum of embryos injected (n_1_), sum of injection survivors to fertile adults (n_2_ or G_0_) and total number of independent transgenic lines created in that species in the compiled Oxitec data set. Accurate as of March 2014

Species	Sum of unique constructs	Sum of embryos microinjected	Sum of microinjection survivors	Sum of independent transgenic lines
*Aedes aegypti*	46	71 252	3314	239
*Aedes albopictus*	10	37 235	5 339	89
*Ceratitis capitata*	26	21 858	5977	167
*Bactrocera oleae*	5	27 500	760	23
*Drosophila suzukii*	4	3287	138	4
*Pectinophora gossypiella*	37	55 605	9296	100
*Plutella xylostella*	34	68 547	21 761	108
*Tribolium castaneum*	2	5227	572	8
*Tuta absoluta*	2	7244	601	3

#### Data entry and checking

A rectangular data set was compiled using data accrued from more than 6 years of research involving the microinjection of nine insect species’ embryos with exogenous DNA.

#### Transformation efficiency distribution

A summary of the data was provided by plotting the injection survival to adulthood against the transformation efficiency for each evaluation unit (Fig. 3B) by insect order. This facilitated comparison to the meta‐analysis as well as Fraser's (2012) description of the *piggyBac* transformation efficiency interpretation. This confirmed the comments of Fraser, with most of the data distributed between 0.01 and 0.1 (117/166 or 70% observations lay within this range).

Interestingly we see a clustering within is clearer, with the Lepidoptera tending to have lower transformation efficiencies (0–0.1) compared to the Diptera, where it is not uncommon to have transformation efficiencies above 0.1. This does not necessarily mean that Lepidoptera are more difficult to transform, as the Lepidoptera injected embryos are more likely to survive. This may be accounted for by differences in injection methodology or by the hardiness of the embryo.

This supports previous work that has compared the variability between efficiencies in the Diptera and Lepidoptera. Lobo *et al*. (2002) compared the mobility of *piggyBac* in embryos from different insect families using a transposition assay. The rate of transposition in dipteran species was higher than that of *T. ni*, which harbours the *piggyBac* transposon (Mohammed & Coates, 2004).

#### Publication bias

For those species for which we have approximately 30 or more experiments with transformation efficiency data, we plotted all the data and highlighted those that have been published (Fig. S8). We observed many more zero and near‐zero transformation efficiencies than would be expected given the meta‐analysis findings hinting at bias (see Supplementary 5.1.1.5 for quantification of the bias). We also observed that atypically high transformation efficiencies tend to be associated with a lower number of injection survivors (quantified in Supplementary 5.1.4.5). This could be caused by researchers stopping their inspection and screening of G_0_ crosses when they feel they have enough lines generated, thereby overestimating the efficiency. Conversely, the lower efficiencies associated with the higher number of injection survivors crossed could be caused by researchers not stopping until they have success. It is also unclear whether all zero successes experiments are recorded.

#### Interspecies variation in survival

**Figure 4:**
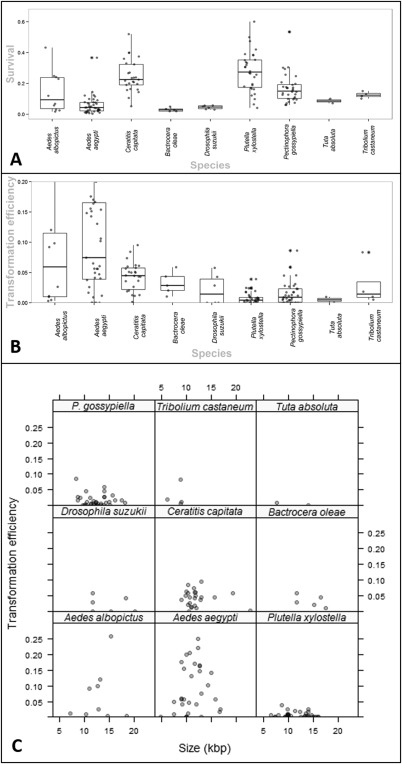
(A) The proportional survival of Oxitec insect research species, from embryo to fertile adult, following microinjection of a *piggyBac* vector. A horizontal jittered scatterplot is overlaid on a boxplot, summarizing the survival distribution for each species. Species are grouped by insect order (Diptera, Lepidoptera and Coleoptera) from left to right. The asterisks show the points that were outliers. For each species there are 10 *Aedes albopictus*, 39 *Aedes aegypti*, 26 *Ceratitis capitata*, five *Bactrocera oleae*, four *Drosophila suzukii*, 29 *Plutella xylostella*, 32 *Pectinophora gossypiella*, two *Tuta absoluta* and two *Tribolium castaneum* data, each representing a unique construct experiment. One outlier at 0.67 survival was removed from *Pl. xylostella* to improve ease of reading. (B). The transformation efficiency of different genetic constructs vectored by *piggyBac* into the germline of different insect species. Transformation efficiency is defined as the number of independent transgenic lines divided by the number of fertile injection survivors crossed, given the unique construct species combination. Few data were found above 0.2 transformation efficiency so the *y* **‐**axis was limited to this range. For each species there are 10 *Aedes albopictus*, 39 *Aedes aegypti*, 26 *Ceratitis capitata*, 5 *Bactrocera oleae*, four *Drosophila suzukii*, 31 *Plutella xylostella*, 35 *Pectinophora gossypiella*, two *Tuta absoluta* and two *Tribolium castaneum* data. (C) A lattice plot of the transformation efficiency of constructs injected into different species by size (in bp). Most constructs are 10 000–15 000 bp in length. As these are injected more frequently there is a cluster of points around this range for each species. Small (< 10 kbp) or large (> 15 kbp) are injected more rarely and so data for these are sparse. The data are subsetted into a species pane with the species label above. Each datum is transparent; dark points represent overlap.

The distributions were visualized using a boxplot and scatterplot hybrid (Fig. 4A). The distributions located further away from the bounds (zero and one) tend to be less skewed and more variable. Extreme values near one or zero are improbable, indeed if zero survival were achieved the results may have been discarded; furthermore, 100% survival does not occur even with uninjected embryos in optimal conditions. Seventeen of the 166 experiments did not include survival data owing to the number of injections or injection survivors missing for the experiment in question. There were no recorded experiments with a zero survival. The maximum survival was achieved in *Plutella xylostella*, with 0.76 compared to the lowest nonzero survival of *Aedes aegypti* at 0.0028.

#### Interspecies variation in transformation efficiency

Most of the data are found between 1 and 10% (Fig. 4B). However, some species appear to be highly clustered, with all *Pl. xylostella* data found between 0 and 5% inclusive. As pointed out by Fraser (2012) the more extreme the transformation efficiencies the less frequently those efficiencies are observed. This can be envisioned as a long tail or a skewed positive distribution. This is observed with the efficiencies far away from the main cluster, as seen in the Diptera and Lepidoptera. For those species with greater than 10 data the interquartile range tends to increase as the median moves away from zero. The tails of both mosquito distributions extends above 20%, with outliers for *Ae. aegypti* as high as 66% (the maximum achieved). Closer inspection reveals the datum responsible comprised of two transgenic lines from three G_0_. This species was described in an earlier study as having a typical transformation efficiency of only 8% (Nimmo *et al*., 2006).

The Lepidoptera have consistently lower transformation efficiencies compared to Diptera. However, both orders have their share of zero transformation efficiency experiments, with 27 in total between them. Those species with lower median transformation efficiency have more zero transformation efficiency experiments. The two Lepidoptera, *Pl. xylostella* (9/31) and *Pectinophora gossypiella* (9/35), have nine each compared to one *Ceratitis capitata* (1/26) and five *Ae. aegypti* (5/39) zero experiments. 19.4% of experiments (27/139) ended without germline transformation, in contrast to the meta‐analysis literature rate of 12% (9/75). This could suggest that those species with lower transformation efficiencies are more likely to have a construct abandoned, or microinjection of DNA is mechanically more difficult, with more injection survivors not containing any plasmid DNA, or simply because of natural variation in the insertion rate.

Despite the nature of the interexperimental variation, such as different constructs injected, different engineers and rearing methods, transformation efficiency is remarkably consistent within some species, particularly the Lepidoptera. This may partly arise as a relic owing to the enforced bounding at zero; however, it does suggest that given a new construct, it is possible to provide a reliable prediction of the transformation efficiency.

As a caveat, some constructs encoded dominant lethal genes as part of a repressible system, and so one might expect a proportion of transgenics to be lost owing to transient (episomal) expression. We might expect this to produce a bi‐modal distribution of transformation efficiency in a species. This may be evident in *Ae. aegypti* (Fig. 4B).

#### Interspecies variation by construct size

Experimental evidence for other transposons suggests a negative correlation between the size of the construct and the transformation efficiency of the vector (Delattre *et al*., 2000). This could be a consequence of larger DNA molecules diffusing smaller distances (Lukacs *et al*., 2000), larger plasmids being injected at a lower molar concentration relative to smaller plasmids or a physical limitation of the vector. Typically a construct injected will be between 10 000 and 15 000 bp in length. It is therefore not recommended to extrapolate the data and try to identify a trend where very few values lie outside the typical range. This is compounded in some species by the low number of constructs injected; each point has a large effect on the overall trend, whereby removal or addition of one datum can change the inference made (Fig. 4C).

Other factors may impact the survival and transformation efficiency of a construct, including operator skill and the coding potential or structure of the construct, for which features such as secondary structure may be important. However, exploration of these factors carries the risk of false‐positives associated with data dredging (Smith & Ebrahim, 2002) and also suffers from limited data and therefore it was not investigated further.

### *Goldilocks* decision‐making: how to get the number of injections just right with *piggyBac*

Injecting too few embryos can result in no or very few transgenic lines. If the lines generated do not show the desired phenotype the investigator is left uncertain as to whether the construct needs to be redesigned or it failed because of position effects. The other extreme involves an excessive number of injections, as the investigator urgently seeks to generate at least one transformed line. Historical data provide an opportunity to estimate the transformation efficiency and guide future experiments.

#### An example using *Pl. xylostella* data

**Figure 5:**
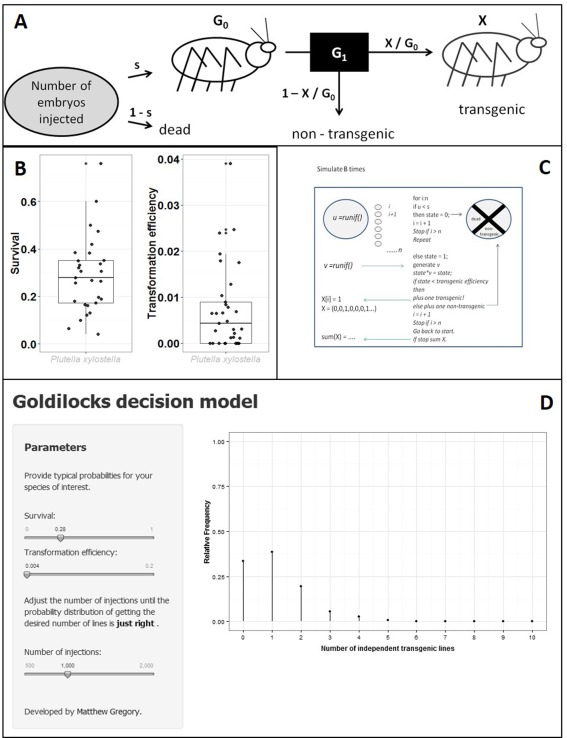
(A) A simple black box model depicting the trajectory of one embryo injected with a novel construct never before injected. The probability that the embryo survives injection, *s*, is based on previous injection data, as is the transformation efficiency, X/G_0_. For a given number of independent injections, *n*, the number of transgenic lines can be estimated given that particular simulation of the model. The stochasticity at each branch adds variability to the output. Accordingly the simulation for *n* injections should be repeated an appropriate number of times to provide a discrete probability distribution of the frequency of transgenic lines produced. (B) A jittered box**‐**and**‐**whisker plot of the survival (*n* = 29) and transformation efficiency (*n* = 33) distributions in diamondback moth (*Plutella xylostella*). The median values are 0.28 and 0.0043, respectively. Outliers are annotated with an adjacent asterisk. (C) A diagram describing the approximate structure of the program used to model the number of independent transgenic lines produced from *n* injections over B simulations. The pseudocode is simplified and does not match precisely how the MATLAB**/**R function works. The reader is advised to start from the top and read from left to right. The variables ‘*s*’ and ‘transformation efficiency’ are input into the model beforehand and are fixed following the example of using the median survival and transformation efficiency. The branching steps provide the stochasticity of the model whereby *u* and *v* are drawn from a random uniform distribution between 0 and 1. These numbers are compared with the input variables, thus determining the fate of the injected embryo. At the first branch it is determined whether the embryo survives (it remains ‘alive’, coded as 0) and at the second whether it gives rise to at least one transgenic offspring. State is a placeholder variable that deals with the previous logic branch by converting the alive embryo to the current value of *v* (multiplying by *v*, if alive state equals *v*, if dead state equals zero). At the end of the loop, the process is repeated, unless the desired injection number has been reached. The number of transgenics is recorded in a vector X and summed upon reaching *n* injects. This provides an integer, which again is stored in a vector of B length. This vector provides the information to draw a discrete probability distribution of the expected number of transgenic lines produced from *n* injections simulated B times. (D) The Goldilocks application interface for helping researchers get the number of injections just right. The output updates when the slider inputs are changed. The most recent data for *Plutella xylostella* are used.

The *Pl. xylostella* survival and transgenesis efficiency are highly skewed, rendering the mean a poor descriptor of the central location of either distribution (Fig. 5B). The median is a better metric as it is more robust to extreme values and because several (9/34) zero values were also present. A representation of the black box model is shown in Fig. 5A. The probability of the embryo failing to achieve G_0_ status is 1 – *s* (where *s* is the median survival of an embryo to G_0_ postinjection). The embryo survives injection, hatches and the larva develops to adulthood with probability *s*. The G_0_ (assumed fertile adult) is then crossed and the offspring G_1_ are screened for the transgene. A G_0_ gives rise to a unique insertion event with probability X/G_0_ (where X is the number of independent lines produced). The published and Oxitec data give the probability of transformation of *Pl. xylostella* under this model as 0.0065 and 0.0043, respectively (Table S4).

#### A decision‐making model for insect transgenesis

A Markov‐Chain Monte‐Carlo simulation (pseudocode in Fig. 5C) was used to model the system in which the number of embryos injected, *n*, gives a binary vector of successes or failures. The final state of each embryo either gives rise to a transgenic‐bearing G_0_ (1) or it does not (0; dead or nontransgenic). Stochasticity is built into the model at each branch where a pseudorandom number (between 0 and 1) is generated and tested against the input parameters as appropriate. The simulation can be run repeatedly to estimate the discrete probability distribution of the total number of transgenic lines produced by *n* injections. The model is named ‘Goldilocks’, to assist researchers in getting the number of injections just right (Fig. 5D). A graphic interface allows the user to adjust sliders to the appropriate values for a species of interest (suggested values are provided in Table 3).

**Table 3:** The median survival and transformation efficiency achieved in species transformed at Oxitec. The statistics in bold are based on many experiments and are probably more reliable. The lowest transformation efficiencies belong to the Lepidoptera

Species	Order	Survival	Transformation efficiency
*Aedes aegypti*	Diptera	**0.093**	**0.059**
*Aedes albopictus*	Diptera	**0.042**	**0.074**
*Bactrocera oleae*	Diptera	0.028	0.029
*Ceratitis capitata*	Diptera	**0.230**	**0.045**
*Drosophila suzukii*	Diptera	0.046	0.014
*Pectinophora gossypiella*	Lepidoptera	**0.149**	**0.009**
*Plutella xylostella*	Lepidoptera	**0.278**	**0.004**
*Tribolium castaneum*	Coleoptera	0.124	0.014
*Tuta absoluta*	Lepidoptera	0.086	0.004

## Conclusion

This paper provides a rigorous description of the distribution of microinjection survival and *piggyBac* transformation efficiencies in different insect species. The paper inspection of the published literature leading on to a detailed exploration and analysis of an unpublished observational data set. This allows insight into publication bias and misconceptions of what is a typical survival or transformation efficiency in a given species. Combined with the Goldilocks decision model, researchers can use this analysis to minimize wasted effort and resources resulting from an inappropriate number of injections being carried out. Owing to the simplicity of assumptions and versatility of the model, Goldilocks can be applied to other germline transformation methods, given that survival and transformation efficiency data are available.

## Experimental procedures

### Meta‐analysis

The following electronic databases were searched from inception to March 2013, repeated in October 2013 and March 2015, to identify relevant experiments and or studies: Web of Knowledge, PUBMED and SCOPUS databases. The key terms used for the search were: piggyBac AND insect*. The database search results were refined by manual inspection and identification of publications with relevant transformation efficiencies. The title and then abstract were read.

Papers were checked for duplicates and removed as appropriate. Each included publication was read by M.G. and data extracted if it met the predefined criteria. If data were missing it was assigned a NA placeholder (NA, not available). The insect species, insect order, unique *piggyBac* construct ID (from the relevant paper to avoid duplication), number of embryos injected, injection survivors and independent transgenic lines derived from those injection survivor crosses were transliterated. The publication search and selection was repeated again 6 months after the initial study selection by the same reviewer and compared (March and October, 2013). Aside from two publications that were newly published, the second search found nine additional relevant publications, possibly because of a more systematic review approach and familiarity with the procedure. Prior to manuscript preparation the search was repeated in March 2015, finding only one new, recently transformed insect. The assumed publication bias will probably have under‐represented the number of failures to transform, as publication of successful transformation of a novel species will tend to be preferred (further details in Supplementary 5.1.1.1).

Data were explored, plotted and modelled using the open‐source R language for statistical computing (R Core Team, 2015). The full data set is available from https://github.com/mammykins/piggyBac-data.

### Oxitec data

The data were transliterated from the original laboratory books as well as student theses (Bilski, 2012; Ant, 2013; Harvey‐Samuel, 2014); contemporary data were also collected from present researchers, and where published cross‐checked against publications. Data were organized in a ‘tidy’ dataframe (Wickham, 2014) and validated by re‐entry. Missing data were treated as described in the Supporting Information (5.1.4.2). The full data set is available from https://github.com/mammykins/piggyBac-data. The observational data were explored, plotted and modelled using R.

### The decision‐making model

The model was initially developed in matlab 2012a Student Version (The MathWorks, Inc., Natick, MA, USA) then re‐coded in R using R studio (http://www.rstudio.com/) and R shiny (http://www.rstudio.com/products/shiny/) to develop a web application for insect transgenesis researchers to use. The model is available online at https://mammykins.shinyapps.io/App-gold and can be implemented locally by using the code from https://github.com/mammykins/Goldilocks-decision-tool.

## Supporting information

Additional Supporting Information may be found in the online version of this article at the publisher's web‐site:

**Figure S1.** A simple method to describe the distribution of the meta‐analysis transformation efficiency values is to plot rank on value. Relative rank is calculated (*p* = r/n) as the proportion of values in the number of trials for the pooled data whose ranks are less than or equal to that value. Percentile information can be retrieved from the figure, for example the median is at relative rank 0.5, upper quartile at 0.75 and lower quartile at 0.25. The rug plot along the *x*‐axis is a uni‐variate scatter of the transformation efficiency estimates pooled from the literature (*n* = 74). Most (∼83%) of the published transformation efficiency data are between 0 and 20%.
**Figure S2.** Funnel plot suggesting bias for transformation efficiency data with confidence interval based on sample size generated using Wilson's method (mean, solid line; 95% confidence interval, dotted and dashed line; 99% confidence interval, dashed line). The plot shows all the nonzero transformation efficiency data collected from the literature (64 nonzero experiments plotted with nine zero experiments; another 13 had missing data).
**Figure S3.** The funnel plot reveals some patient researchers with over 6000 injections in some species before success. Funnel plot of survival data with confidence interval based on sample size generated using Wilson's method (mean, solid line; 95% confidence interval, dotted and dashed line; 99% confidence interval, dashed line). The plot shows all the nonzero transformation efficiency data collected from the literature (64 nonzero experiments plotted with nine zero experiments; another 13 had missing data).
**Figure S4.** In the absence of publication bias we might expect a symmetrical funnel plot. The bounded nature of the proportion data limits the effectiveness of the plot as the Pearson–Klopper confidence intervals are bounded at zero, limiting the size of the 95% confidence interval, which is calculated by subtracting the lower interval from the upper interval at the 95% confidence interval. As the transformation efficiency is not relative to any conventional control, unlike medicine, this removes the relative nature and expected symmetry of the plot.
**Figure S5.** Histogram of the transformation efficiency of the meta‐analysis pooled data with bin width of 0.05. The data are positively skewed and bounded between zero and one. A conventional histogram with associated density curve is shaded grey and a solid line. The area under the curve and between the axes integrates to unity and provides a visual representation of the probability of a transformation efficiency falling in a given interval. The cumulative density histogram is also provided as white bars and a dashed density curve. The graph shows why the use of a mean (0.097 and SD of 0.120) to summarize the data is inappropriate as it is asymmetrical. The bounded nature of the transformation efficiency (between zero and one) also creates problems for the normal approximation.
**Figure S6.** A histogram of the nonzero transformation efficiencies of experiments from the systematic literature review. A beta distribution curve with parameter estimates was overlaid, fitted from the data using the ‘fitdistr’ function from the MASS package in R (Brian Ripley, 1998). The bin width and number of measurements in total forming the histogram were multiplied by the beta distribution so that the curve could be normalized to the correct height. The area under the curve and between the axes integrates to unity and provides a visual representation of the probability of a transformation efficiency falling in a given interval.
**Figure S7.** Bayesian tri‐plot for the mean transformation efficiency probability density in *Tribolium castaneum*. The prior was formulated using 38 transgenics given 95 fertile G_0_ crosses (after Lorenzen *et al*., 2003); the likelihood represents the data of 36 transgenics given 152 fertile G_0_ crosses (after Lorenzen *et al*., 2003). The prior and likelihood are combined using Bayes' theorem to create the posterior distribution, which provides a probabilistic parameter estimate of the transformation efficiency in *Tribolium castaneum* given previous information and recent experimental evidence.
**Figure S8.** The mean and binomial confidence intervals appear to overestimate the transformation efficiency consistently in all four well‐studied species shown (*Aedes aegypti* = 30, *Plutella xylostella* = 33, *Ceratitis capitata* = 26, *Pectinophora gossypiella* = 35). The axes are not constant so care should be taken when comparing between species. The data includes some experiments included in publications highlighted black. The funnel plot provides transformation efficiency data with confidence interval based on sample size generated using Wilson's method (mean, solid line; 95% confidence interval, dotted and dashed line; 99% confidence interval; dashed line). A benefit of the funnel plot is that it highlights the ever‐present danger of mistaking variation owing to chance for correlation or causation.
**Figure S9.** Output from the Goldilocks simulation model – helping researchers to get the number of injections just right. The top and bottom row are simulations involving 500 and 1000 injections, respectively. The left column uses the median statistics from the published data and the right from a more complete Oxitec data set identifying publication bias. The literature provides an overly optimistic view of the chances of successful transformation given a number of injections.
**Table S1.** A timeline of Oxitec publications involving transformation events of an insect species using *piggyBac*. Injection data included number of microinjections, number of injection survivors and number of independent transgenic lines including details of construct and helper concentrations used.
**Table S2.** Only Oxitec in‐house data are summarized (experiments carried out in partnership with Oxitec are not included). An experiment is defined as a unique construct injected into the given insect species. On occasion an experiment will be unsuccessful in that transgenesis is not achieved, described as a zero transgenics experiment. The number of injection survivors crossed and their progeny screened for transgenics varied.
**Table S3.** The probability mass function of the transformation efficiency data found in the literature offers a complete empirical probability mass function version in graphical form. Technically zero is a point not an interval. The probability (0–1) that a transformation efficiency of a publication randomly sampled from the literature sample will fall into given intervals. Convention for 3 significant figures (3.s.f).
**Table S4.** The alpha and beta shape parameter estimates for the fitted beta distribution applied to three different subsets of the Oxitec transformation efficiency data facilitating empirical Bayesian shrinkage towards a beta prior. To utilize these parameters for improving transformation efficiency estimation adjust estimates by: empirical Bayes estimate = (successes + α) / (number of trials + α + β) (Jiang & Zhang, 2010). Prior to subsetting, observations with fewer than 20 injection survivors were removed. Lepidoptera consist of *Plutella xylostella* and *Pectinophora gossypiella*, MedFly just *Ceratitis capitata* and mosquitoes both *Aedes albopictus* and *Aedes aegypti*.
**Table S5.** The diamondback moth parameters to be used in two separate uses of the model to compare differences or the bias produced by reliance on an incomplete data set (published). Both survival and transformation efficiency medians are given for the published and Oxitec data sets.
