# Supplementary material for: Insect transformation with piggyBac: getting the number of injections just right
Source: Insect Mol Biol. 2016 Mar 30;25(3):259–71. doi: 10.1111/imb.12220 (PMC4982070; doi:10.1111/imb.12220)
Supplement: Supplementary file 1 — Figure S1. A simple method to describe the distribution of the meta‐analysis transformation efficiency values is to plot rank on value. Relative rank is calculated (p = r/n) as the proportion of values in the number of trials for the pooled data whose ranks are less than or equal to that value. Percentile information can be retrieved from the figure, for example the median is at relative rank 0.5, upper quartile at 0.75 and lower quartile at 0.25. The rug plot along the x‐axis is a uni‐variate scatter of the transformation efficiency estimates pooled from the literature (n = 74). Most (∼83%) of the published transformation efficiency data are between 0 and 20%. Figure S2. Funnel plot suggesting bias for transformation efficiency data with confidence interval based on sample size generated using Wilson's method (mean, solid line; 95% confidence interval, dotted and dashed line; 99% confidence interval, dashed line). The plot shows all the nonzero transformation efficiency data collected from the literature (64 nonzero experiments plotted with nine zero experiments; another 13 had missing data). Figure S3. The funnel plot reveals some patient researchers with over 6000 injections in some species before success. Funnel plot of survival data with confidence interval based on sample size generated using Wilson's method (mean, solid line; 95% confidence interval, dotted and dashed line; 99% confidence interval, dashed line). The plot shows all the nonzero transformation efficiency data collected from the literature (64 nonzero experiments plotted with nine zero experiments; another 13 had missing data). Figure S4. In the absence of publication bias we might expect a symmetrical funnel plot. The bounded nature of the proportion data limits the effectiveness of the plot as the Pearson–Klopper confidence intervals are bounded at zero, limiting the size of the 95% confidence interval, which is calculated by subtracting the lower interval from the upper interval at the 95% con [file IMB-25-259-s001.docx]

# Insect transformation with *piggyBac*: getting the number of injections just right

Matthew Gregory^1,2^, Luke Alphey^1,2^, Neil I. Morrison^2^ and Sebastian M. Shimeld^1^

1: Department of Zoology, University of Oxford, South Parks Road, Oxford OX13PS, UK

2: Oxitec Ltd, 71 Innovation Drive, Milton Park, Abingdon, OX14 4RQ, UK

3: The Pirbright Institute, Pirbright Laboratory, Ash Road, Pirbright, Surrey, GU24 0NF, UK

## Supplementary methods and analyses

### 5.1.1 Literature Review

#### 5.1.1.1 Data Extraction and Quality Assessment

Publications were inspected for pertinent information. Most transformations with the *piggyBac* vector were achieved in this millennium. Eighty-seven different attempted or realised transformation events were described in the literature. Of these, the majority showed consensus in how to describe the outcomes of the experiment (75 provided complete information detailing injection number, G_0_ and number of transgenic lines). Seven did not include any information about the numbers associated with transformation, asserting that it happened with molecular evidence as support.

#### 5.1.1.2 Types of Outcome Measures

Transformation efficiency was the preferred summary statistic of choice; the number of injection survivors (hereafter termed ‘G_0_’) whose offspring were screened for successful transformation event (independent insertion event, hereafter termed ‘X’). Transformation rate was not provided but could have been calculated from the data provided (number of injected embryos/X, discussed in detail in Adelman *et al*., 2002; Martins *et al*., 2013). Transformation efficiency was the preferred effect size measure (a standard measure by which all outcomes can be assessed) as it controls for the differential mortality rates between species after injection. If any embryo survives to adulthood following injection, what is the probability of it producing transgenic offspring?

#### 5.1.1.3 The distribution of transformation efficiency

Transformation efficiency data allows researchers to design appropriate research methodologies and manage logistics in order to achieve cost effective research. The number of published and included transformation efficiencies were, by insect Order: Coleoptera, seven; Diptera, 62; Hymenoptera, one; and Lepidoptera, 16.

The rugplot elucidates the distribution of transformation efficiencies on a point-by-point basis, showing data points that may be considered outliers or improbable events, such as the transformation efficiency of 66% (Figure S1). On closer inspection this point was shown to be derived from two successes given three trials. Due to the small denominator the confidence interval length will be large compared to other estimates with higher denominator size. Accordingly more robust summary statistics may be preferred to the mean.

If the rugplot of the transformation efficiency data is imagined to be a massless bar and each data point is a weight of equal mass, then the mean represents the moment or fulcrum at which the data is balanced (0.094) whereas the median is the point on the bar which has the same number of weights or data points either side of it (0.004) (n=74). The heavy skew suggests the median is a more appropriate measure of central tendency.


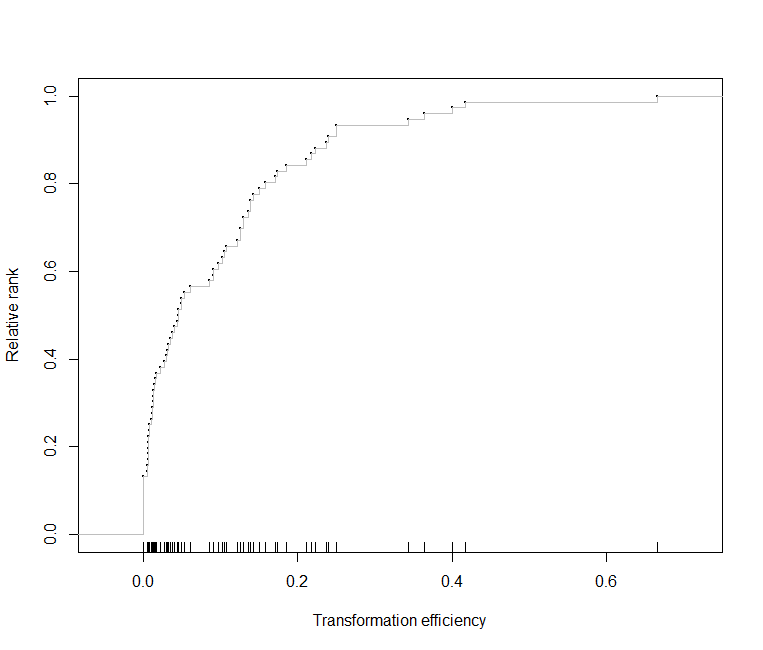


Figure S1. A simple method to describe the distribution of the meta-analysis transformation efficiency values is to plot rank on value. Relative rank is calculated (p = r/n) as the proportion of values in number of trials for the pooled data whose ranks are less than or equal to that value. Percentile information can be retrieved from the figure, for example the median is at relative rank 0.5, upper quartile at 0.75 and lower quartile at 0.25, respectively. The rug plot along the x-axis is a uni-variate scatter of the transformation efficiency estimates pooled from the literature (n=74). Most (~83%) of the published transformation efficiency data is between 0 and 20%.

Cumulative rank scatterplots facilitate understanding where “most” of the data lie (or an empirical cumulative distribution function; Figure S1). Fraser (2012) described transformation efficiencies of 0.001-0.1 as being most probable with efficiencies outside this range less probable. The distribution above suggests that approximately 62% of the data lies between 0-0.1, given a leptokurtic characteristic. However as there are 10 experiments in which no transformants were generated, less than 50% of the data are located in the interval suggested by Fraser (2012). This suggests that our understanding of the distribution of *piggyBac* transformation efficiencies needs refining.

#### 5.1.1.4 Bias considerations

The biased nature of publications should be considered when interpreting data (Dubben & Beck-Bornholdt, 2005; Dwan *et al*., 2013). Data describing a failure to achieve transformation, potentially considered less interesting by the research community, may be omitted from publications or rejected outright. This information, referred to as silent evidence (Taleb, 2007), will produce a systemic error in our estimations of the likelihood that transgenesis will occur.

It is accepted that this review will be subject to publication bias. To what extent it is a problem is typically visualised using a funnel plot (Peters *et al*., 2006). As the experiments become less precise (lower denominator or G_0_ number), it would be expected for the results to be more variable. Interestingly a large number of experiments are close to zero and lie well outside the 99% confidence interval (assuming a binomial distribution, the CI is calculated using the Wilson method which has been shown to be less bad to other methods when dealing with extreme probabilities (near zero or one)). These experiments showed great patience possibly injecting above and beyond what is typical. This may suggest publication bias and that the published experiments overestimate how difficult it is to achieve transformation using *piggyBac*.

The highest transformation efficiencies tend to be in those experiments with fewer number of injection survivors involved in crosses. However, using a mean of all insects injected with *piggyBac* assumes they are one population, the problem of this assumption is compounded by the proximity of the mean to zero which limits the variation at that bound. In the least, the funnel plot (Boessenkool, 2014) provides a caveat to those researchers too hasty to jump to conclusions about the cause of a deviation of an experiment’s transformation efficiency from the mean; it is probably due to chance (Figure S2).
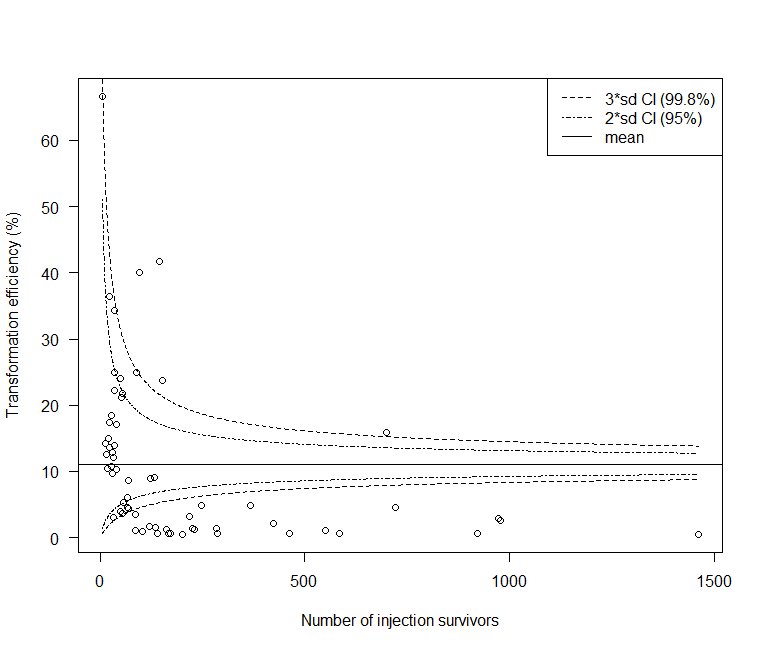


Figure S2. Funnel plot suggesting bias for transformation efficiency data with confidence interval based on sample size generated using Wilson’s method (mean – solid line; 95% confidence interval – dotted and dashed line; 99% confidence interval; dashed line). The plot shows all the non-zero transformation efficiency data collected from the literature (64 non-zero experiments plotted with nine zero experiments; another 13 had missing data).

To reveal the inadequacy of this approach we can compare the suitability of the funnel plot with slightly less skewed data, in the survival data for each experiment (Figure S3). However, this also suffers from our treating each species’ ability to survive microinjection as homogenous.


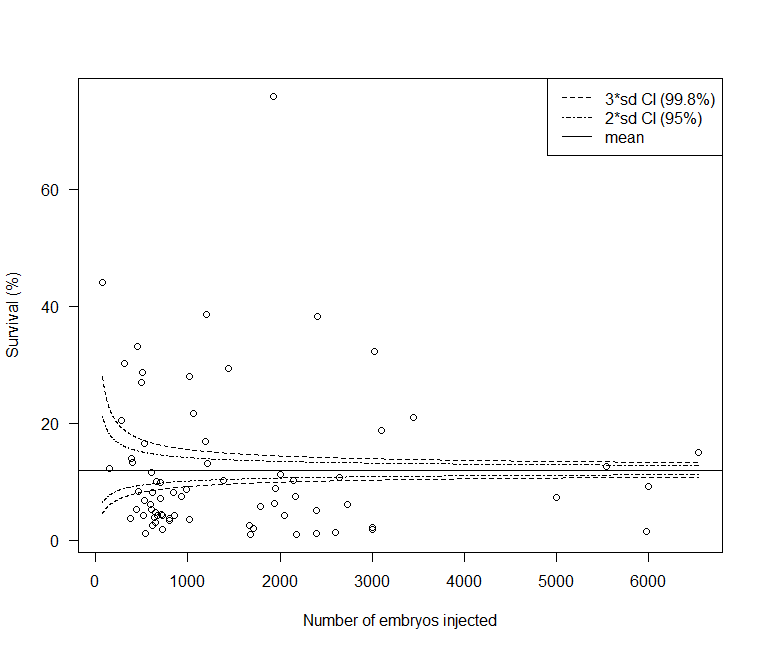


Figure S3. The funnel plot reveals some patient researchers with over 6000 injections in some species before success. Funnel plot of survival data with confidence interval based on sample size generated using Wilson’s method (mean – solid line; 95% confidence interval – dotted and dashed line; 99% confidence interval; dashed line). The plot shows all the non-zero transformation efficiency data collected from the literature (64 non-zero experiments plotted with nine zero experiments; another 13 had missing data).

It would be interesting to apply this approach to a data set that was more complete for a given species and transgenesis marker combination as this would control for inter-species variation. To mitigate these issues one can transform each transformation efficiency data and use the length of the 95% confidence interval calculated for the associated experiment and plot them.


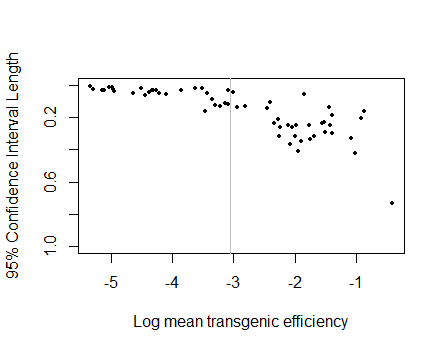


Figure S4. In the absence of publication bias we might expect a symmetrical funnel plot. The bounded nature of the proportion data limits the effectiveness of the plot as the Pearson-Klopper confidence intervals are bounded at zero, limiting the size of the 95% confidence interval which is calculated by subtracting the lower interval from the upper interval at the 95% confidence interval. As the transformation efficiency is not relative to any conventional control, unlike medicine, this removes the relative nature and expected symmetry of the plot.

Figure S4 does not resemble an inverted funnel. This may indicate publication bias; however there are other factors that can lead to an asymmetrical plot. The bounded nature of the proportion data limits the effectiveness of the plot as the Pearson-Klopper confidence intervals are bounded at zero, limiting the size of the 95% confidence interval which is calculated by subtracting the lower interval from the upper interval at the 95% confidence interval. This is borne out by the trend in the figure: as the sample mean efficiency moves away from zero the maximum possible interval size increases. The pooling of species and heterogeneity between research groups may also contribute to the asymmetrical funnel plot. Conventional funnel plots in medicine use a statistic that summarises the treatment effect relative to the control. In this situation no obvious control exists rendering the funnel plot less useful in detecting publication bias.

This bias was not explicitly controlled for in the analysis by statistical corrections. However, the dichotomy between zero and non-zero transformation efficiencies was mentioned and analyses proceeded following sub-setting the data by this criterion.

#### 5.1.1.5 Quantifying bias

The under-reporting of failure to generate transformants could be quantified by comparing a representative sample of all the experiments that have been conducted, and then comparing the differences to the smaller pool that have been published, noting any differences. Comparing Oxitec grey data with publications; the frequency of failures to transform in Oxitec laboratories relative to other published failures to transform were compared.

A time-line of publications by Oxitec researchers is given. Included are details of the number of unique construct and species combinations whether any zero transformation efficiency experiments were included, the name of the construct injected, the publication details and whether precise injection data was given (Table S1). References are ordered chronologically and alphabetically. Publications which re-used a previously published construct species combination were omitted from the list.

Table S1. A timeline of Oxitec publications involving transformation events of an insect species using *piggyBac*. Injection data included number of microinjections, number of injection survivors and number of independent transgenic lines including details of construct and helper concentrations used.

| **Year** | **First Author** | **Species** | **Unique constructs** | **Injection data status** |
| --- | --- | --- | --- | --- |
| 2005 | Gong | *Ceratitis capitata* | 2 | absent |
| 2006 | Dafa’alla | *Ceratitis capitata* | 3 | provided |
| 2006 | Nimmo | *Aedes aegypti* | 1 | provided |
| 2007 | Condon | *Anastrepha ludens* | 2 | provided |
| 2007 | Fu | *Ceratitis capitata* | 2 | absent |
| 2007 | Phuc | *Aedes aegypti* | 1 | provided |
| 2009 | Morrison | *Ceratitis capitata* | 1 | absent |
| 2010 | Fu | *Aedes aegypti* | 2 | absent |
| 2010 | Labbé | *Aedes albopictus* | 1 | provided |
| 2011 | Simmons | *Pectinophora gossypiella* | 1 | absent |
| 2012 | Ant | *Bactrocera oleae* | 1 | incomplete |
| 2012 | Labbé | *Aedes albopictus* | 1 | provided |
| 2012 | Martins | *Plutella xylostella* | 4 | provided |
| 2012 | Morrison | *Pectinophora gossypiella* | 3 | absent |
| 2013 | Jin | *Plutella xylostella* | 2 | absent |

The information above highlights how only non-zero transformation efficiency experiments are likely to be published. The total number of experiments performed at Oxitec permits a true value of the proportion of experiments that result in non-transformation to be compared to an estimate based on the published literature.

Complete information is provided by the columns on the left and compared to the incomplete picture provided by those published experiments (Table S2).

Table S2. Only Oxitec in-house data is summarised (experiments carried out in partnership with Oxitec are not included). An experiment is defined as a unique construct injected into the given insect species. On occasion an experiment will be unsuccessful in that transgenesis is not achieved, described as a zero transgenics experiment. The number of injection survivors crossed and their progeny screened for transgenics varied.

| **Species** | **Number of zero transgenics (all experiments published)** | **Total experiments (all experiments published)** | **Number of zero transgenics (published by Oxitec)** | **Total experiments (published by Oxitec)** |
| --- | --- | --- | --- | --- |
| *Aedes* *albopictus* | 0 | 10 | 0 | 2 |
| *Aedes aegypti* | 5 | 39 | 0 | 4 |
| *Plutella xylostella* | 9 | 31 | 0 | 6 |
| *Ceratitis capitata* | 1 | 26 | 0 | 5 |
| *Bactrocera oleae* | 0 | 5 | 0 | 1 |
| *Pectinophora gossypiella* | 9 | 35 | 0 | 4 |

The published data underestimates the probability of a zero transformation efficiency in three of the five species (no zeroes in those species least injected or most recently experimented on, *Aedes albopictus* and *Bactrocera oleae*). In the Lepidoptera 27% (18/66) of constructs injected as unique experiments failed to produce any transgenic insects. Furthermore almost half of the publications failed to provide detailed injection data to estimate the transformation efficiency of *piggyBac* with that particular species construct combination (7/15). Unsurprisingly the number of total experiments published by Oxitec is much smaller than the number of experiments carried out. The naïve researcher examining the literature may misjudge the probability of success and the number of intermediary constructs required to achieve a final ‘product’ line, or a line that is perceived to be original enough to warrant publication. Further complications may arise due publication being withheld due to patent concerns and intellectual property rights.

Let us assume the position of a naïve researcher interested in creating a *Plutella xylostella* transgenic to elucidate if the subset of the data we are provided with is problematic (published literature). Given no zero transformation efficiencies in six experiments, the mean risk of zero transformation efficiency is zero, but what would the 95% upper confidence level (UCL) be? Using a standard frequentist technique (Ludbrook & Lew, 2009) we estimate the UCL to be 39% (Dorai-Raj, 2014) (LCL of zero). This compares to the empirical probability of a zero transformation efficiency at Oxitec of 29% (9/31).

If we compare totals then standard research at Oxitec has produced zero transformation efficiency constructs 16% of the time (24/146, or 11-23%, 95% Wilson Confidence Interval) yet Oxitec published research never has zero transformation efficiencies. This is compounded by zero transformation efficiencies rarely being reported by other researchers. This could be mitigated by researchers / editors including / requesting all injection data associated with the development of the genetic construct. Information pertaining to which constructs did not work may be useful in modelling predictors of success or failure when developing new constructs (i.e. do larger constructs have a lower transformation efficiency?).

Published constructs are typically developed piecemeal by incremental increases in knowledge by genetic modules or cassettes that can link together to produce a final construct with a desired phenotype. Although this development is interesting in itself, if a construct does not work it is uninformative as to why it does not work and can be difficult to examine the cause of the failure. This may explain the lack of zero transformation efficiency constructs in the literature, purely because they are perceived to be uninteresting and uninformative.

The aim of this section was to draw attention to the publication bias that exists and to attempt to quantify it for researchers interested in using the *piggyBac* vector for their own insect transgenesis research. The reasons for this are likely to be complex and many.

### 5.1.2 Statistical description of the transformation efficiency data

#### 5.1.2.1 Appropriate summary of the transformation efficiency data

A popular summary statistic of the central location of a distribution is the mean, which provides an unbiased estimate (Jones *et al*., 2009). However, it does suffer from some problems when assumptions of symmetry and normality in the distribution break down. To demonstrate why this is inappropriate a histogram of the data was plotted with an overlay of the density and cumulative density distribution curves (Figure S5). The histogram bin width and number of measurements in total were used to normalise the density curves to the correct height.


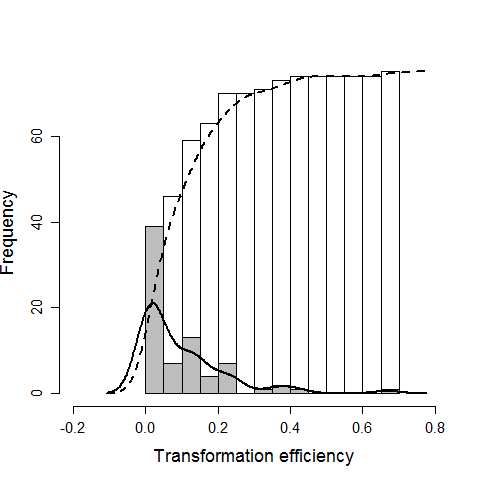


Figure S5. Histogram of the transformation efficiency of the meta-analysis pooled data with bin width of 0.05. The data is positively skewed and bounded between zero and one. A conventional histogram with associated density curve is shaded grey and a solid line. The area under the curve and between the axes integrates to unity and provides a visual representation of the probability of a transformation efficiency falling in a given interval. The cumulative density histogram is also provided as white bars and a dashed density curve. The graph shows why the use of a mean (0.097 and standard deviation of 0.120) to summarise the data is inappropriate as it is asymmetrical. The bounded nature of the transformation efficiency (between zero and one) also creates problems for the normal approximation.

The bounded nature of the data and the heavy skew means the normal distribution is a very poor model of the data. The skew and leptokurtosis render the mean useless as a descriptive statistic of the central location of the transformation efficiency distribution in insects injected with *piggyBac* vector.

Assuming history can be used to predict the future (ignoring the problem of induction) the expected value of the transformation efficiency of a random construct can be described. For the moment we ignore any effects of differences between the experimental conditions from which the transformation efficiencies were derived and assume that transformation efficiency of a *piggyBac* vector is described by a mean efficiency and a random error term.

The data can be modelled discretely by using intervals of transformation efficiency. The table below gives the probability mass function for transformation efficiency of constructs published in the literature (Table S3). It summarises the probability P(y) of achieving a transformation efficiency in a given interval, the discrete random variable (y).

Table S3. The probability mass function of the transformation efficiency data found in the literature offers a complete empirical probability mass function version in graphical form. Technically zero is a point not an interval. The probability (0-1) that a transformation efficiency of a publication randomly sampled from the literature sample will fall into given intervals (3.s.f).

| Transformation efficiency interval (y) | 0 | >0-0.1 | >0.1-0.2 | >0.2-0.3 | >0.3 |
| --- | --- | --- | --- | --- | --- |
| P(y) | 0.135 | 0.486 | 0.230 | 0.095 | 0.068 |

The empirical cumulative distribution function (ECDF) can be calculated and plotted for the transformation efficiency data (to convert to percentages multiply by one hundred). It provides all of the information from the data in a graphical format which can be easily read from Figure S1. For example if a researcher wants to estimate the probability that a transformation efficiency greater than 0.2 will be achieved, one would trace up from p=0.2 and then along to the associated f(p) value of 0.8 (it can also be computed given the ECDF; a re-mapping of the data). This tells us that approximately 0.8 of all observations are below a transformation efficiency of 0.2, therefore the fraction of observations greater than 0.2 transformation efficiency is only (1-0.8) approximately 0.2 of the observations.

Transformation efficiency is a continuous variable; accordingly it would be preferable to fit a probability density function with a suitable mathematical model to describe the data; compressing the data into a function of two numbers. The model could then be used to describe probabilities with given values of the transformation efficiency by integration of the curve.

It can be difficult to arrive at a decision as to what distribution best characterises the data, as normally one would make a decision based upon the data distribution using a histogram. The problem with the histogram is that the interval size of the bins can affect the geometry of the distribution. An alternative approach is to consider how the transformation efficiency value is generated and its mathematical properties, and consult the literature for strategies to model a fit to the data.

Conventionally for proportion data the logit transformation was used to normalise the distribution of proportion data. Different probabilities of success, p, are transformed into logit (p) (or log (p/1-p)). As there are a large number of zeroes, this may not be appropriate as the logit of zero is minus infinite. Adding one to all data causes additional problems. The logit transformation can also make interpretation difficult so an alternative strategy was preferred.

#### 5.1.2.2 Probability density function of the transformation efficiency data

Transformation efficiency is a derived variable. It is assumed to be emergent from a series of independent and identically distributed random variables (X1, X2, X3... Xn; range: success (transgenic) =1, failure (non-transgenic) = 0), with constant probability of success p.

Transformation efficiencies of zero or one were removed from the data and a subset created of transformation efficiencies with “no zeroes”. This subset provides us with a strategy to generate a probability density function of the probability of obtaining a transformation efficiency value on condition that transgenesis was achieved (transformation efficiency is non-zero, p > 0 and < 1). According to Table S3, the probability of achieving a non-zero transformation efficiency is 0.865 (1 - 0.135).


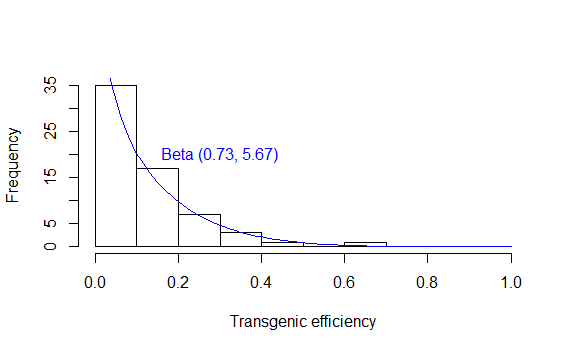


Figure S6. A histogram of the non-zero transformation efficiencies of experiments from the systematic literature review. A beta distribution curve with parameter estimates was overlaid, fitted from the data using “fitdistr” function from the MASS package in R (Brian Ripley, 1998). The bin width and number of measurements in total forming the histogram were multiplied by the beta distribution so that the curve could be normalised to the correct height. The area under the curve and between the axes integrates to unity and provides a visual representation of the probability of a transformation efficiency falling in a given interval.

This distribution function can be used to estimate the probability of achieving transformation efficiencies in any specified interval using the base “pbeta” function in R with the shape 1 and shape 2 parameters for the beta distribution derived from the data using maximum likelihood methods (Figure S6). It is a more convenient and succinct version of the ECDF, as it summarises the information into a function of two numbers. This information is useful for decision-making and informing researchers as to the likely number of fertile injection survivor crosses required for success. This number can then be multiplied by the injection-related mortality in the species of interest to determine an appropriate number of injections required for successful transformation (given the data or prior information in literature). This may facilitate estimating research costs. The beta distribution format of the probability density function is amenable to use in Bayesian analyses and decision-making, increasing the utility of these findings (for a technical description see Jiang & Zhang, 2010).

Going back to Fraser (2012); using this model the probability of the non-zero transformation efficiency of a *piggyBac* vector insertion lying between 0.001 and 0.1 is 0.586; most efficiencies are in this interval when the data is summarised using a beta distribution.

To summarise - if a researcher is working with a species for which no literature is available to suggest a transformation efficiency estimate, then Figure S6 provides a distribution of the expected transformation efficiency given past successes in insects, dependent that the species is amenable to transformation and the construct can indicate successful integration (and the construct is non-lethal).

### 5.1.3 Bayesian analysis of transformation efficiency – a toy example with *Tribolium castaneum*

#### 5.1.3.1 Tribolium castaneum transformation efficiency estimate from several experiments

Although the variation between experiments is large it seems a plausible range of likely values of the transformation efficiency can be observed for a species which has seen numerous independent experimental instances of transformation particularly if the same promoter-fluorescent protein combination were used to report success. The *Tribolium castaneum* transformation efficiency lies between 0.2-0.4 making it amenable to Bayesian analysis. Of course this subjective interpretation should be revised and objective methods based on meta-analysis strategies of combining summary statistics from different experiments should be employed. This information can be used to produce a prior transformation efficiency estimate or preferably a probability distribution of likely transformation efficiencies for a particular species given enough experiments. This could then be used as a decision tool for estimating the number of G_0_ required for likely success in *Tribolium castaneum*.

The intended outcome is an appropriate probability density function for use in Bayesian analysis. An approximate answer to the right question is desired, over a precise answer to the wrong question (the frequentist approach would not permit prediction of a parameter associated with an event that has not yet happened, O’Hagan & Luce, 2003). A controversial assumption includes that differences in the design of the constructs have negligible impact on transformation efficiency (based on the same transgenesis module being used within each species). The data is derived from the same lab so methodologies are broadly similar with some discrepancies in plasmid and helper concentrations, for example.

#### 5.1.3.2 Bayesian analysis for a Bernoulli process

An example of how to merge transformation efficiency data for a single species from different experiments will be demonstrated using a Bayesian approach (O’Hagan, 2009) following methods of Winkler *et al*., 2002. The use of a prior distribution and the Bayesian approach is robust even in the face of the zero-numerator problem (Ludbrook & Mew, 2009), providing excellent coverage in situations likely to occur in insect transgenesis research.

For consistency, the methods will be described using the symbols in Winkler *et al*., (2002). For a more detailed discussion of the technique, the reader is referred the details in this paper (the original approximation for the binomial process using the beta distribution used in Winkler is after Ishii and Hayakawa (1960)).

Imagine you were in the position of Lorenzen *et al*., in 2003. In an earlier experiment they had successfully transformed *Tribolium* *castaneum* using *piggyBac*. They generated 38 transgenic lines with 95 G_0_. Going into the experiment they have an estimate of the transformation efficiency in *Tribolium castaneum* based on the maximum likelihood estimate of the mean. The Bayesian approach requires a prior distribution which can be estimated conveniently:

*f(p) = p^a-1^(1-p)^b-1^/B(a,b) for 0 ≤ p ≤ 1*

The estimate of the mean is *a / (a+b)* with *a* the number of successes and *b* the number of failures. The true mean, *p*, is the probability of success bounded between 0 and 1.

The information about *p* represented by the prior Beta( *a, b* ) distribution can be interpreted as the equivalent of having seen *a* transgenics given *a + b* fertile injection survivors crossed.

Given the prior, in a later experiment, if we observe *r* independent transgenic lines given *n* trials (the data is the likelihood) the posterior distribution is Beta( *a + r, b + n – r* ).

*Prior*: after Lorenzen (2003), a = 38, b = 95.

The prior was chosen based on the only relevant empirical data available at the time.

*Likelihood*: after Lorenzen (2003), r = 36, n = 152.


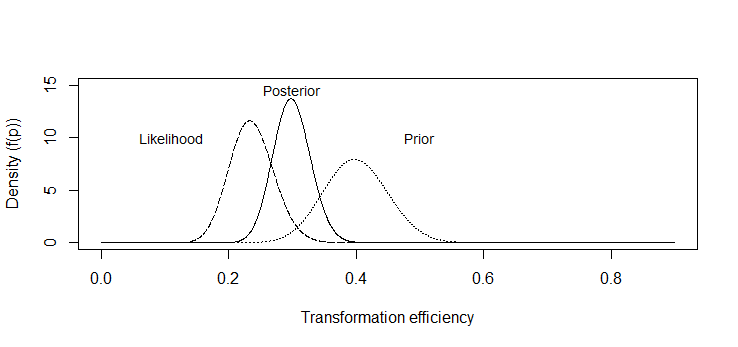


Figure S7. Bayesian tri-plot for the mean transformation efficiency probability density in *Tribolium castaneum*. The prior was formulated using 38 transgenics given 95 fertile G_0_ crosses (after Lorenzen *et al*., 2003), the likelihood represents the data of 36 transgenics given 152 fertile G_0_ crosses (after Lorenzen *et al*., 2003). The prior and likelihood are combined using Bayes’ Theorem to create the Posterior distribution which provides a probabilistic parameter estimate of the transformation efficiency in *Tribolium castaneum* given previous information and recent experimental evidence.

Bayes’ theorem allows us to combine information from two different sources using explicit methodology. The resulting posterior distribution permits inferences about the parameter of interest; the transformation efficiency of *piggyBac* in *Tribolium castaneum*. Observational studies can provide strong inference for parameter estimation but are limited in assessing causality.

It follows that the transformation efficiency is described by the posterior probability distribution with the mode at 0.298. This provides an intuitive probability distribution providing plausible parameter estimates with the integral of the distributions. The reliability of the estimate is based on the similarity between the experiments and methodology, as well as assumptions of the binomial model of insect transgenesis success described above.

### 5.1.4 Oxitec data

#### 5.1.4.1 Structure

The term data set (or dataframe) defines a set of measurements taken from some environment or process. The data set will take the form of the collection of measurements on *n* objects, comprising the rows (unique constructs injected into a unique species). The columns are made up of *p* variables which represent the columns and variables of the data set; which has size, *n* times *p* (after Little & Rubin, 2002). The data was represented as a dataframe in R, with missing values assigned “NA”, for not available, missing values were due to a failure to observe or record (Adler, 2010). The data is comprised of empirical distributions of variables, based on a finite number of empirical observations that will continue to expand and update through time. A well structured data frame lends itself to continual updating facilitating future analysis and hypothesis generation by interested researchers.

#### 5.1.4.2 Dealing with missing values

In the data set some of the entries were not observed (constructs could be abandoned before success rates were measured or entries were not made and or not recorded, etc.). These values are missing in the sense that there were actual values that could have been assigned to the entry if observation or recording techniques were not lacking. These entries were replaced with a suitable place holder given the statistical software used for analysis (NA when using R; Adler, 2010). After Little & Rubin (2002) it is assumed that “missingness indicators hide true values that are meaningful for analysis”.

One simple way around the problem of missing values is to exclude them from the analysis. This strategy is termed “complete-case analysis”. Although straightforward this approach can be problematic given a small data set or the nature of the missingness, known as the missing data mechanism. If the missing data mechanism has some dependence on the values of the data the mechanism is called “not missing at random” (NMAR). The missing data from our data-set seems to be NMAR. As suggested earlier this is due to the nature of the research environment; if a very similar construct has been very successful in generating transgenics (or in generating transgenics that do not have the predicted or desired phenotype) observations were not made. Data were transliterated chronologically, assuming they were entered originally in a chronological order, based on this assumption the missingness of data seems to be clustered around certain time periods.

This NMAR makes it difficult to estimate missing values. Accordingly complete-case analysis was preferred. Following removal of all rows any NA or NaN (not a number, for example when dividing by zero) value in any column 149 complete entries or rows of the 166 remained each described by the five variables (species, construct ID, injections, injection survivors and number of lines generated) of interest for this study (27 variables were included for posterity). Due to these differences, systematic error between missing and non-missing data may occur, confounding analysis. The probability that an observation is missing is likely to be dependent on the outcome being studied.

This was too conservative; ideally any entry would be removed if it tested NA given the specific analysis being carried out (i.e. for a particular explanatory variable involved in the analysis). This approach, called pairwise deletion, was preferred.

#### 5.1.4.3 The experimental unit and the evaluation unit

The experimental procedure for generating transgenics is summarised in Figure 3A, and was kept constant except for species type of embryo and the construct injected. The species of the embryo forced experimental constraints on variables that could be controlled including rearing practices and ratios at which the injection survivors were pooled with wild type insects to give rise to G_1_. The nomenclature used is based on suggestions of Hurlbert (2009): an experimental unit is defined as “The smallest system or unit of experimental material to which a single treatment … is assigned … and which is dealt with independently.”

The construct label itself is a random factor. The size of the construct injected and presence or absence of certain genes were also considered during analyses. Each trial (or experimental unit) (n) is summarised in the master data frame (https://github.com/mammykins/piggyBac-data). Each row or observation in the data set comprised a large number of independent repeats of “n” (minimum = 100, median = 1480, maximum = 12000) or injections. The data set is comprised of almost 297,755 experimental units which provide numerical, random categorical, fixed categorical and derived variables on 166 observations.

#### 5.1.4.4 The number of microinjections and methodological considerations

The Diptera have received 161,132 embryo microinjections between them (see Manuscript Table 2). This is split between two teams or departments at Oxitec: one specialising in transformation of fruit flies, the other in mosquitoes. Due to challenges in embryo collection and preparation compared to the Lepidoptera, the injections tend to be conducted by teams of people rather than individual researchers, with regular swapping of roles to maintain motivation and concentration throughout (the moth transformation team carried out embryo collection and injection as individuals). It is unclear how random this process was and how much variation a researcher carrying out the injections has on this data exploration. Methodologies were similar throughout the collection of the data as all participants sought process optimisation by careful control of variables and monitoring or recording of any changes to methods. Importantly the marker modules (promoter and fluorescent protein to indicate successful germline transformation) were relatively constant within a species.

Almost 131,400 lepidopteran (pink bollworm, diamondback moth and *Tuta absoluta*) embryo injections made up the Lepidoptera data sub-set. Of these, 211 produced unique insertion events that were detected over the 6 years.

The Coleoptera despite being the most speciose order are relatively neglected with only 5,000 embryo injections in *Tribolium castaneum*.

#### 5.1.4.5 Bias considerations when comparing the meta-analysis and Oxitec data

Cross referencing the transformation efficiency data against the number of injection survivors used showed a correlation between the highest transformation efficiencies and lowest number of injection survivors; the experiments with the lowest number of trials produced higher efficiencies, as shown in the funnel plots (Figure S8). This provides an important caveat to consider when inferring a trend from this data.

Some of the Oxitec experiments have been published with the summary statistics included in the meta-analysis data. There published experiments are compared to all other experimental data for that species transformation efficiency at Oxitec (Figure S8). The skew also reinforces that the mean is a poor measure of central tendency for transformation efficiency with the median preferred.


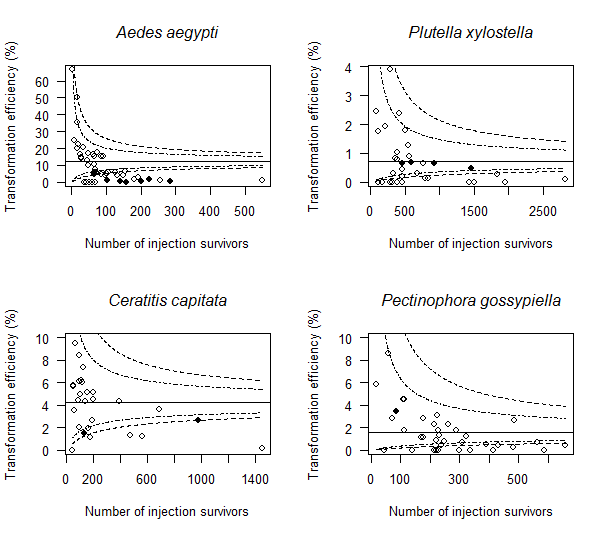


Figure S8. The mean and binomial confidence intervals appear to overestimate the transformation efficiency consistently in all four well-studied species shown (*Aedes aegypti* = 30, *Plutella xylostella* = 33, *Ceratitis capitata* = 26, *Pectinophora gossypiella* = 35). The axes are not constant so care should be taken when comparing between species. The data includes some experiments included in publications highlighted black. The funnel plot provides transformation efficiency data with confidence interval based on sample size generated using Wilson’s method (mean – solid line; 95% confidence interval – dotted and dashed line; 99% confidence interval; dashed line). A benefit to the funnel plot is that it highlights the ever present danger of mistaking variation due to chance for correlation or causation.

Given the problem of estimating a large number of proportions for each construct and species combination the statistical method of empirical Bayes estimation was used (Jiang & Zhang, 2010). In the meta-analysis we demonstrated how using the beta distribution to represent your prior expectations, and updating based on the new evidence, can help make an estimate more accurate and practical (5.1.3). Here we use the Beta distribution to correct a large set of estimates controlling for the uncertainty in cases with low number of injection survivors crossed. This involved three steps, first we sub-setted the data into appropriate groups based on the EDA; the Lepidoptera, MedFly and the mosquitoes (some species were omitted entirely). For each group an overall Beta distribution was fitted (using the fitdistr function in the MASS package in R, Venables & Ripley (2002)), with each relevant distribution used as the prior for estimating each empirical-Bayes-corrected transformation-efficiency-estimation (Table S4).

Table S4. The alpha and beta shape parameter estimates for the fitted Beta distribution applied to three different subsets of the Oxitec transformation efficiency data facilitating empirical Bayesian shrinkage towards a Beta prior. To utilise these parameters for improving transformation efficiency estimation adjust estimates by: empirical Bayes estimate = (successes + α) / (number of trials + α + β) (Jiang & Zhang, 2010). Prior to sub-setting observations with fewer than twenty injection survivors were removed. Lepidoptera consist of *Plutella xylostella* and *Pectinophora gossypiella*, MedFly just *Ceratitis capitata* and mosquitoes both *Aedes albopictus* and *Aedes aegypti*.

| **Group** | **α** | **β** |
| --- | --- | --- |
| Lepidoptera | 1.3 | 84.4 |
| MedFly | 2.2 | 47.7 |
| mosquitoes | 1.2 | 12.7 |

#### 5.1.4.6 Survival data considerations

As the median of each distribution approaches 0.5 the variance appears to increase while the skew decreases, suggesting a normal approximation could be viable for the distribution of a samples survival in *Plutella xylostella* and *Pectinophora gossypiella*. Conversely those species distributions with a nearer zero median may be better represented by a non-normal distribution.

Three of the species have outliers – constructs injected that were more than 1.5 times the inter-quartile range (IQR) away from the median. Outliers occur in those species with a greater number of data; as they are simultaneously improbable and extreme. For those species with smaller IQR it is easier to predict a future expected survival following microinjection with greater accuracy. Those four species with the lowest IQR were also the four species with the lowest number of constructs injected and should be excluded from the previous assertion.

Walton *et al*., 1987 showed a relationship between the diameter of the needle and the probability of embryo lysis or cellular disintegration although this was not consistent between species. It was speculated that this was due to the relative size of the embryo to the needle and the taper of the needle. The different species at Oxitec are injected with needles made from different programme specifications on a glass-capillary needle puller (programmes have changed through time). Despite this confounding variable, it would be interesting to compare species embryo average dimensions to needle diameter ratio with embryo survival.

Recent additions to the Oxitec research portfolio have fewer experiments and lower variation (Figure 4A; specifically *Tuta absoluta, Tribolium castaneum*, *Bactrocera oleae* and *Drosophila suzukii*). Those with more injections tend to have greater variation. This could reflect improved injection and husbandry systems for working with the particular insect species through time (or the natural tendency for the range of a rare event statistic to increase through time). Interestingly the mosquitoes have been injected for many years but still show a low survival of typically less than 10% this suggests there may be a ceiling to survival for microinjection.

#### 5.1.4.7 Considerations for the decision model

The more variable the survival the less useful it is to ask – given n injections how many transgenic lines can one expect? For these species it may be preferred to ask – given a number of G_0_, how many transgenic lines can one expect? This was considered an important requisite of a decision model. Accordingly the survival can be set to one to assess the transformation efficiency given a number of G_0_.

It is also important to note that the decision model was constructed in a way to make it useable to other researchers (they provide fixed input parameters based on their empirical evidence or information in this review) and should not be limited by potential lab-specific biases that relying on only the Oxitec data could create. For species for which many prior experiments exist it may be preferred if the survival and transformation efficiency of each simulated embryo experiences a parameter value drawn from the empirical distribution weighted by the size of the denominator for that species. This approach was investigated but sacrificed speed for precision.

The inclusion of an average fertility rate in the model was omitted as this information is typically unavailable to most researchers. The default model assumes full fertility of injection survivors. This could be adjusted if the relevant information is available by transforming the transformation efficiency. For example, if it is known that half of one hundred injection survivors are fertile then the transformation efficiency would be the number of transgenics produced divided by fifty, not one hundred.

#### 5.1.4.8 Assessing decision making using incomplete information

The model was run for 500 injections and 1000 (top and bottom rows of Figure S9) injections for both the published data and Oxitec data parameters (left and right columns).

Table S5. The diamondback moth parameters to be used in two separate uses of the model to compare differences or the bias produced by reliance on an incomplete data set (published). Both survival and transformation efficiency medians are given for the published and Oxitec datasets.

|  | **Published** | **Oxitec** |
| --- | --- | --- |
| **Survival (s)** | 0.3839 | 0.2800 |
| **Transformation efficiency** | 0.0065 | 0.0043 |

The parameters derived from the literature reveal the survival estimate lies outside the inter-quartile range of our complete survival data set. The transformation efficiency estimate compares more favourably and is close to the median derived from the complete data set (Figure S9).


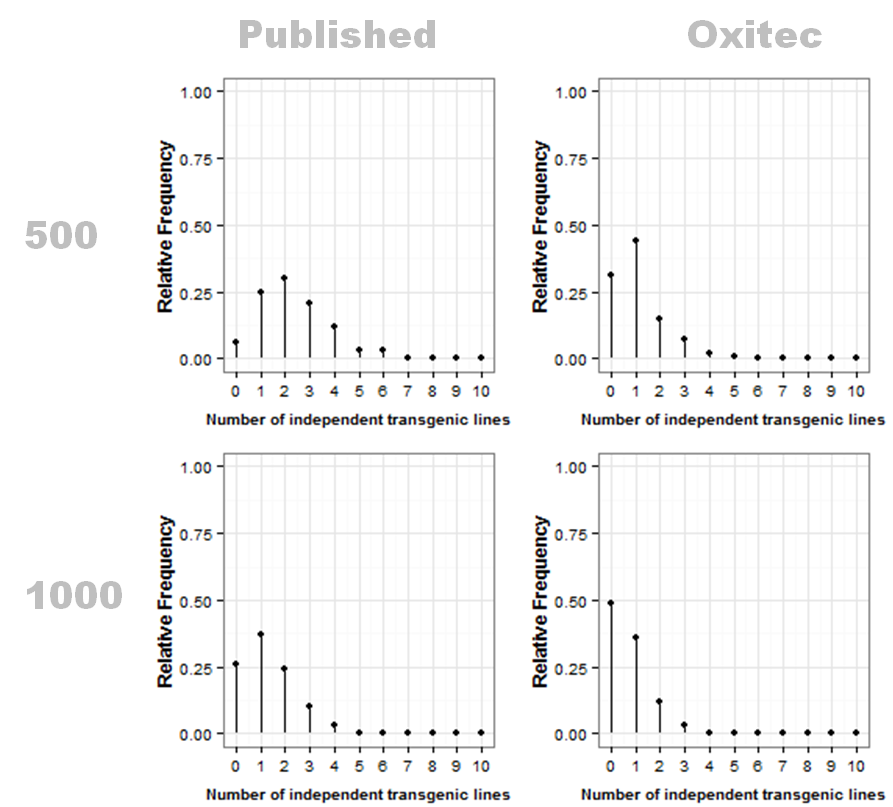


Figure S9. Output from the goldilocks simulation model – helping researchers to get the number of injections just right. The top and bottom row are simulations involving 500-1000 injections, respectively. The left column uses the median statistics from the published data and the right from a more complete Oxitec data set identifying publication bias. The literature provides an overly optimistic view of the chances of successful transformation given a number of injections.

The relative frequency is the number of simulations that resulted in the specified number of transgenic lines divided by the total number of simulations. The model demonstrates how basing ones decisions on published data could lead to a failure to transform due to insufficient injections. The simulations contrast with 500 injections whereby the published data suggests a 25% chance of failure compared to a 50% chance using the complete data set. Doubling the workload to a thousand injections almost halves the probability of failure for both the published and complete data set parameters.

This approach applied to other well-documented species at Oxitec revealed the literature overestimates the transformation efficiency for the Lepidoptera (*Plutella xylostella* and *Pectinophora gossypiella*) and underestimates for *Aedes aegypti* and *Ceratitis capitata*. The moral is to use as complete a dataset as possible to ensure a better understanding of what is typical.

#### 5.1.4.9 Applying the model

Download R, R studio, the Shiny and ggplot2 packages in R and the Goldilocks application. Run the application. Carry out the number of injections necessary to reduce the risk of zero transgenic lines to an acceptable relative frequency. This model should be used as a heuristic and should not replace thinking about the specifics of the relevant problem a researcher faces. Alternatively an online version is available at https://mammykins.shinyapps.io/App-gold.

The model has many uses and can be used in similar situations for other vectors or even other modes of transformation, such as the gene gun. If summary statistics are available for the survival and transformation efficiency, then this can be co-opted for other methods and facilitate decision making for gene insertion approaches.

This model can also be used to provide a p value for justifying when one has carried out sufficient injections for a particular construct.

#### 5.1.4.10 A preferred model using all the data at the expense of time to simulate

The previous model used two parameter estimates to drive the model; the median survival and the median transformation efficiency. Given many experimental results we can improve precision by using all experimental results. This is achieved by the model drawing from a vector containing all experimental results with the probability of sampling a particular datum weighted by the number of trials used in that particular experiment. This approach was preferred as it used all empirical data available but was not skewed by experimental parameter estimates based on a small number of trials. This provides extra precision at the expense of time to run the simulation. Accordingly for a live web-based application the earlier method was preferred due to a faster computing time. Comparing simulation outputs did not reveal a difference for those insects with many data. For those insects with few data the models are likely to vary in their output (data not shown).

## 5.2 References

Adelman, Z.N., Jasinskiene, N. & James, A. a, 2002. Development and applications of transgenesis in the yellow fever mosquito, *Aedes aegypti*. *Molecular and biochemical parasitology*, 121(1), pp.1–10. Available at: http://www.ncbi.nlm.nih.gov/pubmed/11985858.

Adler, J., 2010. *R in a nutshell* First. M. Loukides, ed., Sebastopol: O’Reilly Media Inc.

Ant, T. et al., 2012. Control of the olive fruit fly using genetics-enhanced sterile insect technique. *BMC biology*, 10(1), p.51. Available at: http://www.pubmedcentral.nih.gov/articlerender.fcgi?artid=3398856&tool=pmcentrez&rendertype=abstract [Accessed July 21, 2014].

Boessenkool, B., 2015. BerryFunctions package in R. Available at: https://github.com/brry/berryFunctions.

Condon, K.C. et al., 2007. Germ-line transformation of the Mexican fruit fly. *Insect molecular biology*, 16(5), pp.573–80. Available at: http://www.ncbi.nlm.nih.gov/pubmed/17894556.

Dafa’alla, T.H. et al., 2006. Transposon-free insertions for insect genetic engineering. *Nature biotechnology*, 24(7), pp.820–1. Available at: http://www.ncbi.nlm.nih.gov/pubmed/16823373 [Accessed July 21, 2014].

Dorai-Raj, S., 2014. Package “binom”: Binomial Confidence Intervals For Several Parameterizations. Available at: http://cran.r-project.org/web/packages/binom/index.html.

Dubben, H. & Beck-Bornholdt, H., 2005. Systematic review of publication bias in studies on publication bias. *BMJ (Clinical research ed.)*, 331(7514), pp.433–434. Available at: http://www.pubmedcentral.nih.gov/articlerender.fcgi?artid=1188108&tool=pmcentrez&rendertype=abstract [Accessed May 29, 2013].

Dwan, K. et al., 2013. Systematic review of the empirical evidence of study publication bias and outcome reporting bias - an updated review. *PloS one*, 8(7), p.e66844. Available at: http://www.pubmedcentral.nih.gov/articlerender.fcgi?artid=3702538&tool=pmcentrez&rendertype=abstract [Accessed November 4, 2013].

Fraser, M.J., 2012. Insect transgenesis: current applications and future prospects. *Annual review of entomology*, 57, pp.267–89. Available at: http://www.ncbi.nlm.nih.gov/pubmed/22149266 [Accessed August 31, 2012].

Fu, G. et al., 2010. Female-specific flightless phenotype for mosquito control. *Proceedings of the National Academy of Sciences of the United States of America*, 107(10), pp.4550–4. Available at: http://www.pubmedcentral.nih.gov/articlerender.fcgi?artid=2826341&tool=pmcentrez&rendertype=abstract [Accessed June 11, 2011].

Fu, G. et al., 2007. Female-specific insect lethality engineered using alternative splicing. *Nature biotechnology*, 25(3), pp.353–7. Available at: http://www.ncbi.nlm.nih.gov/pubmed/17322873 [Accessed September 13, 2011].

Gong, P. et al., 2005. A dominant lethal genetic system for autocidal control of the Mediterranean fruitfly. *Nature biotechnology*, 23(4), pp.453–6. Available at: http://www.ncbi.nlm.nih.gov/pubmed/15750586 [Accessed October 19, 2011].

Hurlbert, S.H., 2009. The ancient black art and transdisciplinary extent of pseudoreplication. *Journal of comparative psychology (Washington, D.C. : 1983)*, 123(4), pp.434–43. Available at: http://www.ncbi.nlm.nih.gov/pubmed/19929111 [Accessed October 4, 2012].

Ishii, G. & Hayakawa, R., 1960. On the compound binomial distribution. *Annals of the institute of statistical mathematics*, 12(1), pp.69–80.

Jiang, W. & Zhang, C., 2010. Empirical Bayes in-season prediction of baseball batting averages. *Institute of Mathematical Statistics Collections*, 6, pp.263–273.

Jones, O., Maillardet, R. & Robinson, A., 2009. *Introduction to Scientific Programming and Simulation using R*, Boca Raton, Florida: Chapman & Hall.

Kongmee, M. et al., 2010. Irritant and Repellent Behavioral Responses of Aedes aegypti Male Populations Developed for RIDL Disease Control Strategies. *Journal of Medical Entomology*, 47(6), pp.1092–1098. Available at: http://www.bioone.org/doi/abs/10.1603/ME10046 [Accessed October 7, 2011].

Labbé, G.M.C. et al., 2012. Female-specific flightless (fsRIDL) phenotype for control of Aedes albopictus. *PLoS neglected tropical diseases*, 6(7), p.e1724. Available at: http://www.pubmedcentral.nih.gov/articlerender.fcgi?artid=3393675&tool=pmcentrez&rendertype=abstract [Accessed November 28, 2012].

Little, R. & Rubin, D.B., 2002. *Statistical Analysis with Missing Data*, John Wiley & Sons Ltd., Hoboken, New Jersey.

Lorenzen, M.D. et al., 2003. piggyBac-mediated germline transformation in the beetle Tribolium castaneum. *Insect molecular biology*, 12(5), pp.433–40. Available at: http://www.ncbi.nlm.nih.gov/pubmed/12974948.

Ludbrook, J. & Lew, M.J., 2009. Estimating the risk of rare complications: is the “rule of three” good enough? *ANZ journal of surgery*, 79(7-8), pp.565–70. Available at: http://www.ncbi.nlm.nih.gov/pubmed/19694672 [Accessed August 20, 2012].

Martins, S. et al., 2012. Germline transformation of the diamondback moth, Plutella xylostella L., using the piggyBac transposable element. *Insect Molecular Biology*, 44(0), p.no–no. Available at: http://doi.wiley.com/10.1111/j.1365-2583.2012.01146.x [Accessed May 24, 2012].

Morrison, N.I. et al., 2012. Engineered Repressible Lethality for Controlling the Pink Bollworm, a Lepidopteran Pest of Cotton. *PloS one*, 7(12), pp.1–10.

Morrison, N.I. et al., 2009. Sexual competitiveness of a transgenic sexing strain of the Mediterranean fruit fly, Ceratitis capitata. *Entomologia Experimentalis et Applicata*, 133(2), pp.146–153. Available at: http://doi.wiley.com/10.1111/j.1570-7458.2009.00909.x [Accessed October 30, 2011].

O’Hagan, A., 2009. Bayesian principles. In *Bayesian Methods in Health Economics: a short course, The Biomedical & Life Sciences Collection*. London. Available at: http://ezproxy.ouls.ox.ac.uk:4492/bio.

O’Hagan, A. & Luce, B., 2003. *A primer on Bayesian statistics in health economics and outcomes research*, MEDTAP International, Inc. Available at: http://scholar.google.com/scholar?hl=en&btnG=Search&q=intitle:a+primer+on+Bayesian+statistics+in+health+economics+and+outcomes+research#0 [Accessed May 14, 2013].

Peters, J. et al., 2006. Comparison of Two Methods to Detect Publication Bias in Meta-analysis. *JAMA: the journal of the American*, 295(6), pp.676–680. Available at: http://scholar.google.com/scholar?hl=en&btnG=Search&q=intitle:Publication+Bias+in+Meta-analysis#0 [Accessed December 2, 2013].

Phuc, H.K. et al., 2007. Late-acting dominant lethal genetic systems and mosquito control. *BMC biology*, 5, p.11. Available at: http://www.pubmedcentral.nih.gov/articlerender.fcgi?artid=1865532&tool=pmcentrez&rendertype=abstract [Accessed July 21, 2014].

Simmons, G.S. et al., 2011. Field performance of a genetically engineered strain of pink bollworm. *PloS one*, 6(9), p.e24110. Available at: http://www.pubmedcentral.nih.gov/articlerender.fcgi?artid=3172240&tool=pmcentrez&rendertype=abstract [Accessed October 21, 2011].

Taleb, N.N., 2007. Black Swans and the Domains of Statistics. *The American Statistician*, 61(3), pp.198–200. Available at: http://www.tandfonline.com/doi/abs/10.1198/000313007X219996 [Accessed March 7, 2013].

Tan, A. et al., 2013. Transgene-based, female-specific lethality system for genetic sexing of the silkworm, Bombyx mori. *Proceedings of the National Academy of Sciences*, pp.1–5. Available at: http://www.pnas.org/cgi/doi/10.1073/pnas.1221700110 [Accessed April 9, 2013].

Venables, W. & Ripley, B., 2002. *Modern Applied Statistics with S* 4th ed., New York: Springer. Available at: http://www.stats.ox.ac.uk/pub/MASS4.

Walton, J.R. & Marshall, J.T., 1987. Zygote Viability in Gene Transfer. *Biology of reproduction*, 37, pp.957–967.

Winkler, R., Smith, J. & Fryback, D., 2002. The Role of Informative Priors in Zero-Numerator Problems : Being Conservative Versus Being Candid. *Statistical Practice*, 56(1), pp.1–4.
